# Improving workflow control in radiotherapy using discrete-event simulation

**DOI:** 10.1186/s12911-019-0910-0

**Published:** 2019-10-24

**Authors:** Bruno Vieira, Derya Demirtas, Jeroen B. van de Kamer, Erwin W. Hans, Wim van Harten

**Affiliations:** 1 Department of Radiation Oncology, Netherlands Cancer Institute, Antoni van Leeuwenhoek Hospital, Amsterdam, Plesmanlaan 121, 1066 CX Amsterdam, The Netherlands; 2 Center for Healthcare Operations Improvement and Research (CHOIR), University of Twente, Enschede, The Netherlands; 3 Department of Industrial Engineering and Business Information Systems, Faculty of Behavioural Management and Social Sciences, University of Twente, PO Box 217, 7500 AE Enschede, The Netherlands; 4 Department of Health Technology and Services Research, School of Governance and Management, University of Twente, PO Box 217, 7500 AE Enschede, The Netherlands; 5 Rijnstate General Hospital, Arnhem, The Netherlands

**Keywords:** Workflow control, Radiotherapy, Discrete-event simulation, Resource planning, Waiting times

## Abstract

**Background:**

In radiotherapy, minimizing the time between referral and start of treatment (waiting time) is important to possibly mitigate tumor growth and avoid psychological distress in cancer patients. Radiotherapy pre-treatment workflow is driven by the scheduling of the first irradiation session, which is usually set right after consultation (pull strategy) or can alternatively be set after the pre-treatment workflow has been completed (push strategy). The objective of this study is to assess the impact of using pull and push strategies and explore alternative interventions for improving timeliness in radiotherapy.

**Methods:**

Discrete-event simulation is used to model the patient flow of a large radiotherapy department of a Dutch hospital. A staff survey, interviews with managers, and historical data from 2017 are used to generate model inputs, in which fluctuations in patient inflow and resource availability are considered.

**Results:**

A hybrid (40% pull / 60% push) strategy representing the current practice (baseline case) leads to 12% lower average waiting times and 48% fewer first appointment rebooks when compared to a full pull strategy, which in turn leads to 41% fewer patients breaching the waiting time targets.

An additional scenario analysis performed on the baseline case showed that spreading consultation slots evenly throughout the week can provide a 21% reduction in waiting times.

**Conclusions:**

A 100% pull strategy allows for more patients starting treatment within the waiting time targets than a hybrid strategy, in spite of slightly longer waiting times and more first appointment rebooks. Our algorithm can be used by radiotherapy policy makers to identify the optimal balance between push and pull strategies to ensure timely treatments while providing patient-centered care adapted to their specific conditions.

## Background

Radiotherapy (RT) is a therapy modality for cancer treatment that requires several preparation steps consisting of imaging and treatment planning. RT resources are expensive and limited in capacity, and treatments are prepared and delivered by a multidisciplinary group of specialists with multiple activities and limited time availability [1]. As demand for RT continues to grow [2] and cancer treatments become more personalized [3], ensuring a timely delivery of RT for each patient trajectory without jeopardizing the timeliness of the other patients is not straightforward. Earlier research has shown that the dynamic nature of treatment scheduling in RT, in which scheduled and non-scheduled patients have to be queued up for undergoing pre-treatment, can considerably impact access times for RT [4, 5]. Long waiting times^1^ have been associated with negative clinical outcomes such as higher risk of local recurrence [6], increased tumor progression [7] and prolonged psychological distress in patients [8]. In fact, the unavailability of medical staff was pointed out as one of the main causes for this [9]. Related to this, Hutton et al. found that RT professionals in the UK are prone to the effects of compassion fatigue and burnout and that special attention must be paid to workload and its impact on practitioners’ job satisfaction [10].

The RT treatment process starts with referral, followed by a consultation with a radiation oncologist, who prescribes the necessary steps needed (referred to as “pre-treatment workflow”) before the treatment starts. The pre-treatment workflow includes imaging (CT, MRI, PET-CT), contouring of the tumor and organs-at-risk, and treatment planning, and is commonly driven by the scheduling of the first irradiation session, which is usually set immediately after consultation. This demands pre-treatment workflow to be programmed a priori before the scheduled starting date for treatment. We refer to this strategy as the “pull” strategy [5], a term derived from logistics and supply chain management where manufacturing is driven by customer demand and resources are expected to be available at each operation when needed for just-in-time production. In RT, a pull strategy foresees that a date for the start of treatment is set right after consultation, and the scheduling of pre-treatment workflow is performed in a “backwards” fashion, ensuring that the necessary rooms and staff will be available when needed to meet timeliness targets. However, for some patient types, the first irradiation is scheduled after (some) the pre-treatment steps have been completed, typically at the start or at the end of treatment planning This is referred to as “push” strategy, which in logistics terms refers to a continuous flow of products throughout the system, with no specific due date, typically leading to store inventory. By applying a push strategy in radiotherapy flexibility to perform pre-treatment activities and consequently a low number of first linac appointment rebooks can be expected. However, setting a treatment start date right after consultation (pull strategy) may lead to increased patient and staff (doctors) satisfaction, particularly when time slots for doctors’ activities (e.g. contouring of the tumor) are pre-allocated in coordination with treatment scheduling decisions. It may also increase control over the work in progress, leading to a reduced number of patients breaching the waiting time targets. Therefore, appropriate workflow management systems (e.g. scheduling routines) and the design of efficient resource planning schemes are crucial to meet the intended waiting time targets [11] while ensuring patient centeredness and quality of labor.

Operations research (OR) methods have been successfully used to support decision-making in health care in general [12], and increasingly in radiotherapy [13]. Among OR methods, discrete-event simulation (DES) stands out as a powerful tool to find logistical interventions for performance improvement by modeling the behavior of complex systems as a series of discrete events occurring over time [14]. DES has been proven useful in testing operational changes in several healthcare settings [15], such as analyzing optimal discharge rates in acute care [16], capacity management and patient scheduling in outpatient clinics [17], and decreasing throughput times for CT scanning in radiology departments [18, 19]. In the field of radiotherapy, a few DES studies have been conducted for process improvement and resource planning. Kapamara et al. [20] performed a patient flow simulation analysis to find bottlenecks in the Arden Cancer Center, UK, to reduce waiting times and maximize patient throughput. The authors were able to model three treatment modalities (conventional external-beam, brachytherapy, and unsealed sources therapy), and found that an extension of clinical shift hours reduces patients’ waiting times by 2%. Proctor et al. [21] modeled patient care pathways from arrival to discharge to estimate the impact of increased levels of demand in the performance of the department of RT of the Walsgrave hospital, UK. They reported that reducing the percentage of patients seeing their own doctor on the simulator from 71 to 35% and extending the linacs’ operating hours by 38% would provide the best performance, with 82% of the patients starting treatment within the desired target. Werker et al. [22] used DES as an attempt to improve the RT planning process of the RT center of the British Columbian Cancer Agency in Canada, finding that reducing delays associated with the oncologists’ tasks would reduce the planning times by 20%. Babashov et al. [23] included the treatment stage of the RT trajectory, thus modeling the process from patient arrival to treatment completion. They found that adding one more full-time oncologist would reduce the waiting times by 6.55%, leading to around 85% of the patients starting treatment within 14 calendar days. Crop et al. [5] studied an alternative workflow control system for robotic stereotactic RT by testing a constant work-in-progress system that only allows new patients to start pre-treatment when a patient leaves the system, in an attempt to keep workload constant. Results showed that a hybrid constant work-in-progress workflow could potentially increase the number of irradiation sessions per day by 32%, while the time between CT and start of treatment remained stable at an average of 9 days.

Computer simulation studies of RT are available but mainly focus on finding operational improvements by re-dimensioning workforce, expanding machine capacity/availability, or extending clinical opening times, whilst the impact of implementing alternative scheduling routines and different workflow control systems are rarely found. In this work, we model the RT pre-treatment workflow using DES to quantify the operational impact of using pull and push strategies in RT scheduling. As a secondary goal, we try to find interventions (e.g. increase treatment planning capacity) that maximize the number of patients starting treatment within the intended targets and allow for minimal waiting times.

## Methods

We used DES modeling to construct a model on the flow of patients receiving external-beam RT in the Netherlands Cancer Institute (NKI) from consultation to the start of treatment (first fraction). The model was built using Tecnomatix Siemens Plant Simulation 13.2 by Siemens PLM Software [24]. After the model was validated, we studied the impact of increasing the number of pull patients starting from the baseline case representing the current practice (40% pull / 60% push), as well as other possible interventions for performance improvement.

### The RT treatment workflow in the NKI

**Fig. 1:**
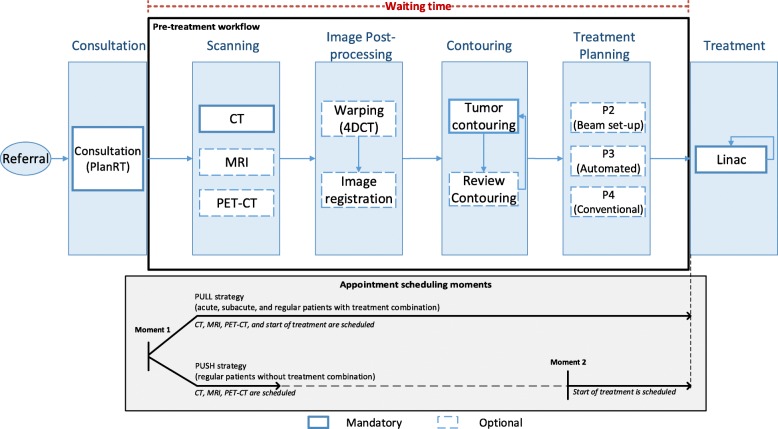
Flowchart of the complete RT treatment workflow in the NKI

Figure 1 depicts the RT workflow in the NKI. Upon referral, patients are scheduled for a consultation (Moment 1) with a radiation oncologist, who becomes responsible for monitoring the patient’s care trajectory. At consultation, the doctor meets the patient and assesses all the information needed to plan an RT treatment. After consultation, the doctor fills in a form (PlanRT) with the medical information and sets up a preliminary treatment plan outlining the care pathway intended for the patient. The pre-treatment workflow starts after consultation, when patients are scheduled for a CT scan, but a delay before pre-treatment starts, due to other appointments (e.g. IV-contrast, blood analysis, manufacturing of patient-specific aids such as masks etc.) may be needed, as well as additional imaging examinations (MRI and PET-CT). In case a 4DCT has been taken, imaging motion compensation is needed (warping). If multiple imaging scans are involved, then the registration of the different datasets is also necessary (image registration). Thereafter, the doctor delineates the target area (contouring), right before treatment planning. At this step, beam set-up (simplified treatment planning such as the two-field technique “anterior-posterior-posterior-anterior”) may be done instead or in conjunction with regular treatment planning. Once treatment planning is finished, the generated plan is uploaded to the corresponding linac and the treatment can start. The modeled pre-treatment workflow, indicated by the black bounding box in Fig. 1, starts right after consultation (PlanRT) and ends at the start of treatment. The time needed to complete the pre-treatment phase is referred to as “waiting time” in this study.

Regarding the appointment scheduling process, Fig. 1 shows that upon submission of the PlanRT sheet after consultation, an appointment officer schedules all the necessary imaging scans for all patients. This moment in time is represented by “Moment 1” in Fig. 1. At Moment 1, acute patients, subacute patients, and regular (i.e. non-urgent) patients who have a combination of RT with other treatment modality (surgery or chemotherapy) are also scheduled for all irradiation sessions right after consultation. We refer to these as “pull” patients. Acute and subacute patients are scheduled in a pull manner as a timely start of treatment needs to be ensured due to the urgency of their treatment. Regular patients with a treatment combination between RT and other treatment modality (e.g. chemotherapy or surgery) also need to be scheduled right at consultation. For these patients, a proper time coordination between irradiation sessions and the other treatment modality is necessary to maximize the effectiveness of the combined treatment. For pull patients, pre-treatment activities need to be given enough time to be completed before the pre-scheduled starting date to avoid linac sessions’ rebooks. Alternatively, regular patients without a combination of treatment modalities, indicated as “push” patients in this study, are scheduled for the start of treatment only once contouring has been done and treatment planning has started, as indicated by Moment 2 in Fig. 1.

### Model inputs

In DES, a number of inputs are needed to generate events (e.g. patient arrivals, processing times, resource availability) that represent the behavior of the real system. In our model, we used historical data from the whole year 2017 (January 01 to December 31) as model inputs to (randomly) generate those events. To obtain data that was not available in the internal databases, we conducted several interviews with radiation oncologists, radiation therapy technologists (RTTs), managers and appointment schedulers to estimate the most realistic values for each input parameter. Table 1 presents an overview of all input parameters of our DES model.

**Table 1:** Input parameters of the DES model

Name	Description	Probability Distribution	Dependencies
Patient arrivals	Patient arrival rates per weekday, per tumor site (8 independent generators)	Poisson	–
Care plan	Proportion of patients in each of the 62 possible care trajectories depending on tumor site (generators)	Empirical	Tumor site
Urgency level	Proportion of acute, subacute, and regular per care plan	Empirical	Care plan
Steps needed	Proportion of patients with CT, MRI, PET-CT, warping, image registration, contouring, and treatment planning type, per care plan, per urgency level	Empirical	Care plan Urgency level
CT/MRI/PET-CT processing times	CT = 25 min. MRI = 45 min, and PET-CT = 45 min, regardless of other parameters.	–	–
Image Post-processing times	Mean and standard deviation of the duration for processing warping and image registration.	Lognormal	–
Contouring time	60 min for tumor contouring, and 60 min for peer-review review.	–	–
Treatment planning times	Processing times of P2, P3, and P4, depending on the care plan.	–	Care plan
Scheduling of first fraction	Proportion of patients for each possible duration of the time-to-treatment (0 … 21 days) per urgency level, per weekday	Empirical	Weekday Urgency level
Planned delay	Proportion of patients with a planned delay before pre-treatment, and the length of the delay (1 … 8 weeks), per care plan	Empirical	Care plan
Machine availability	Time of the day each CT, MRI, and PET-CT is available to be operated	–	–
Doctors’ agenda	Start time and end time for each day of the simulation period, and parts of the day the doctor is unavailable due to other scheduled activities (meetings, research, etc.)	–	–
RTTs’ agenda	Start time and end time for each day of the simulation period	–	–
Public holidays and days-off	Days of the simulation period in which the clinic is not operating, and days each RTT and doctor is unavailable (days-off)	–	–

### Model development

**Fig. 2:**
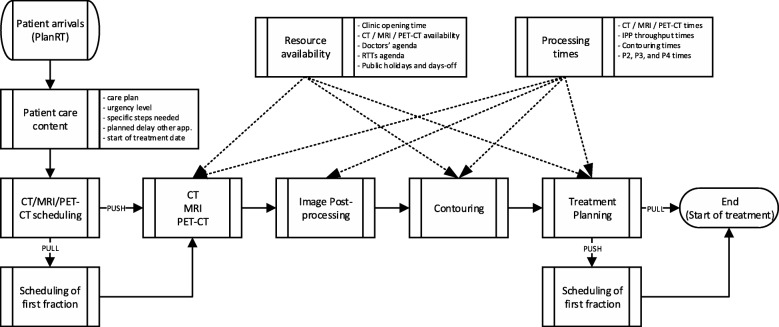
Components of the DES model and their relations with input parameters

The modeled steps, scheduling routines and their relationship with the input parameters are depicted in Fig. 2. The specific workflow and data contained in each component are explained in more detail throughout this section. Patient arrivals are generated using records of PlanRT form creation dates (after consultation), followed by the creation of patient care content according to the probability distributions mentioned in Table 1. At this point, push patients will be scheduled the necessary imaging scans, and will proceed to the pre-treatment workflow CT/MRI/PET-CT/IPP, contouring and treatment planning. Pull patients will also be scheduled the start of treatment before following the same route. The start of treatment of push patients is then scheduled at treatment planning. “Resource availability” and “processing times” contain the logistics data used in the scanning, contouring, image post-processing, and treatment planning steps.

#### Patient arrivals

We used historical data from the year 2017 to determine probability distributions for the arrival processes in the NKI, which are used in the DES model to generate patient arrivals. We considered the historical records of all PlanRT forms filled in by the doctors after consultation as patient arrivals, excluding weekends and public holidays. In total, we included 4973 patient care pathways recorded in 2017 for external-beam RT treatments. Earlier research has shown that there were statistically significant differences in the patients arrivals between workdays, and that patient arrival patterns follow a Poisson distribution in each workday [25]. An updated ANOVA analysis with the 2017 data using the probability-distribution fitting software EasyFit [26] resulted in the same conclusions (Table 2), i.e. patient arrivals were found to follow a Poisson distribution for every weekday.

**Table 2:** Patient arrival statistical analysis for the 2017 data

Weekday	Sample Size	Prob. Dist.	Mean (SD)	*p*-value
Monday	859	Poisson	17.5 (4.7)	0.72
Tuesday	1067	Poisson	20.9 (5.7)	0.24
Wednesday	1208	Poisson	23.2 (6.7)	0.61
Thursday	1063	Poisson	21.7 (5.9)	0.51
Friday	776	Poisson	15.5 (5.4)	0.25

**Fig. 3:**
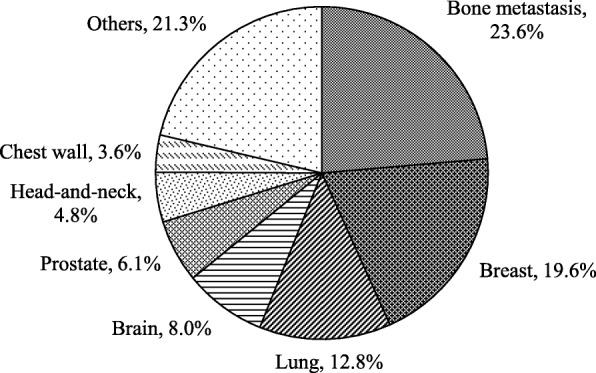
Distribution of patients by tumor site in 2017

In the NKI, patients are assigned one of eight possible tumor sites upon referral: Bone metastasis, Breast, Lung, Brain, Prostate, Head-and-neck, Chest wall, or Others, as depicted in Fig. 3. Each tumor site has a different consultation pattern over the week. For instance, consultations for (regular) lung patients are mostly held on Wednesday mornings. Therefore, we generated patient arrivals in the model by using the mean arrival rate per tumor site, per weekday, according to a Poisson distribution (Table 2) and using the proportions presented in Fig. 3.

#### Patient care content

The attributes of each patient (care plan, urgency level, specific steps needed, planned delay before pre-treatment, and start of treatment date) were randomly assigned based on the historical breakdown measured in 2017. After consultation, the doctor selects one of 62 possible trajectories for the patient, which depends on the tumor site for that patient (see Additional file 1). For instance, a lung patient may be assigned the palliative trajectory, or the regular trajectory, which would yield a different care pathway. The care trajectory defines whether a patient would require MRI (18.5% of the population), PET-CT (3.9%), Warping (12.4%), Image registration (29.7%) or Beam set-up (34.7%). All patients require a CT, contouring, and treatment planning. The urgency level indicating whether a patient is acute (1.3% of the patient population), subacute (30.8%), or regular (67.9%) was generated based on the historical proportions verified for the corresponding trajectory. Moreover, measured data shows that 650 out of the 4973 patients (13%) have a planned delay before starting pre-treatment (CT) due to medical reasons (e.g. RT after surgery, dentist) or patient preferences (e.g. holidays), the delay ranging between 1 and 8 weeks. In the 2017 data, we found that 40.8% of the patients were scheduled in a pull fashion (SD = 5.8%), while the remainder 59.2% were scheduled using on a push fashion. Empirical distributions using the above-mentioned proportions were used to create patient care content in each replication of each computational experiment.

#### CT/MRI/PET-CT scheduling

Scheduling of scanning appointments in imaging rooms are assigned on a first-come-first-planned basis, except for some appointments in CT scanners, where a pre-allocation of specific time slots exists. For instance, the first two time slots in the morning cannot be assigned to patients who need IV-contrast before the CT, as the corresponding doctor must be present in the department but may not have started his/her shift before 08 h30. Similarly, there is one time slot exclusively available for acute patients per day.

#### Contouring

Doctors are grouped in teams based on their specialty: Breast, Lung, Urology, Head-and-neck, Gynecology, Gastrointestinal tract, and Central nervous system. Table 3 presents the total number of doctors per specialty. Depending on the specific tumor site, a doctor belonging to the corresponding specialty is assigned to the patient using empirical distributions from the 2017 data. Contouring of palliative patients (acute and bone metastasis), accounting for 815 of the 4973 patients, can be undertaken by any available doctor right after scanning. Pending contouring activities waiting in queues are sorted on an Earliest Due Date (EDD) basis, giving priority to the patients with the earliest date for start of treatment. For push patients, who have not been scheduled at this point, we considered the target date for start of treatment according to the national targets.

**Table 3:** Doctor teams and corresponding number of elements in the NKI during 2017

Specialty	No. doctors
Lung	7
Head-and-neck	9
Breast	9
Central nervous system	3
Gynecology	4
Gastrointestinal tract	5
Urology	7
Palliative	All (44)

#### Treatment planning

Treatment planning is divided in three types: P2, P3, and P4. There used to be a P1 type that does not currently exist in the NKI. P2, also referred to as beam set-up, is a simpler form of planning mostly undertaken for bone metastasis and some breast cancer patients. P3 is a form of automated planning in which a computer software performs the planning autonomously. P4 is the conventional treatment planning modality, in which beam angles and intensities are iteratively optimized with the help of a computer software. P3 is immediately assigned to all breast, rectum, and prostate patients, as the planning of these tumor sites was automated in 2017. P4 will be assigned to all patients belonging to the other patient groups who have not been assigned P2 or P3. The assignment of P2 is modeled by means of empirical distributions that vary per care plan, i.e. the probability of a patient being assigned P2 varies depending on the care plan of that patient (see Additional file 1). For instance, 93% of all bone metastasis patients will have a P2 type of planning, while a head-and-neck patient will never be assigned P2, which means that he/she will always be assigned P4. Out of the 24 planning RTTs available, 3 hold a P2 level, 7 are skilled at level P3, and the remaining 10 are considered at level P4. P4 planners are also able to perform P3 and P2, and P3 planners can also perform P2. Moreover, P3 and P4 level planning RTTs can process 2 plans simultaneously. As with the previous step, treatment planning of acute patients and bone metastasis patients can be performed by any available planner right after scanning, and queued tasks are prioritized on an EDD basis.

#### Scheduling of first fraction

A statistical analysis showed that the time between arrival and the start of treatment do not follow any specific probability distribution with sufficient statistical significance (*p*-value > 0.05). Therefore, we used empirical distributions to randomly assign a date for start of treatment for both pull and push patients. For pull patients (40% of the total population), a treatment start date is generated based on the historical records upon first consultation. Since certain care plans have starting date requirements (e.g. head-and-neck patients must start on a Monday), we generated this time to treatment depending on the weekday of the request. This means that, for instance, a regular head-and-neck patient having the first consultation on a Tuesday will most likely be assigned a time to treatment of 6 or 13 days. According to the measured data, time to treatment of pull patients ranges between 0 and 1 day for acute patients, between 1 and 8 days for bone metastasis and subacute patients, and between 3 and 21 days for regular patients. Push patients (60%) are assigned a time between treatment planning and start of treatment that can range between 1 and 7 days, also generated on a weekday basis.

#### Resource availability

The RT department of the NKI operates from 07 h30 to 17 h30 on every weekday except public holidays. Staff members work 8 or 9-h shifts (with breaks) while rooms and machines are available during the 10-h period. The department has 2 CT scanners, 1 MRI scanner, and 1 PET-CT scanner. The PET-CT scanner is shared with the diagnostics department. In total, there are 26 time slots of 25 min available per day for CT scanning, 37 weekly slots of 45 min for MRI, and 5 weekly time slots of 45 min for PET-CT. As for staff members, the department hosts a total 113 RTTs (75 FTE), of which 24 can do treatment planning. In addition, there are 44 practitioners (26 FTE) in the department, which include radiation oncologists, residents and physician assistants. Their main duties include patient consultations, regular meetings (such as multi-disciplinary, RT treatment discussions, and research) and other administrative tasks. In the NKI, a doctor is available to perform contouring whenever he/she is not scheduled to do any of the pre-allocated tasks. Except for scheduled activities, the doctor gives priority to perform contouring over the other non-scheduled duties. The weekly schedule and absent days (incl. Holidays, sick leave, conferences, training, etc.) of each staff member throughout 2017 have been used for the staff availability of our model.

#### Processing times

A CT scan has a time slot duration of 25 min, while an MRI and a PET-CT usually take approximately 45 min each. We included two possible tasks (warping and image registration) for IPP based on the historical records, which were found to follow a lognormal distribution with the mean and standard deviation presented in Table 4. If warping is needed for a patient, a delay corresponding to the time between CT and warping (CT-Warping) is generated. In case a patient needs multiple scans and thus has the need for image registration, we forced a delay respective to the time between the last scan (warping included) and image registration (Scanning-Image registration).

**Table 4:** Statistical analysis of IPP tasks: processing times for both CT-Warping and Scanning-Image registration follow a lognormal distribution (*p-*value > 0.05)

Time	Sample Size	Prob. Dist.	Mean (SD)	*p*-value
CT – Warping	608	Lognormal	0.4 (0.6)	0.35
Scanning-Image registration	1306	Lognormal	0.1 (1.0)	0.60

In the NKI, a contouring typically takes up to 30 min for acute and subacute patients, and 1 hour for regular patients to be completed according to the interviewed doctors. Moreover, each contouring needs to be peer-reviewed and approved by another doctor before the process moves on to treatment planning. In the NKI this step is done right after contouring, with the doctor in charge asking a colleague to double check the contouring on site. This extra step takes at most 60 min. Therefore, we have added 60 min to the processing time of each contouring to account for the peer-review task. Standard processing times for beam set-up and treatment planning vary considerably per care trajectory, ranging from 60 (e.g. bone metastasis) to 120 (e.g. breast) minutes for a beam set-up, and from 150 (e.g. prostate) to 960 (e.g. head-and-neck) minutes for treatment planning.

### Model verification

The model was built iteratively in constant interaction with managers and clinicians from the RT department of the NKI. Components of the model as described in “model inputs”, such as patient arrivals generators, staff management tools, and processing units were added step by step after conducting interviews with the staff members of the NKI responsible for that step. The scheduling routines and simplifications introduced in each process were carefully discussed and approved by the manager in charge of the corresponding process.

### Performance metrics

The most important Key Performance Indicators (KPIs) to evaluate the performance of our model are related to timeliness: the waiting times (in calendar days) and the percentage of patients breaching the waiting time targets. Maximum waiting time targets defined by the Dutch Society for Radiation Oncology [11] state that acute patients should be treated within 1 day, subacute patients should start treatment within 10 calendar days, and regular patients should start treatment within 28 days. In addition, we also look at the percentage of first fraction rebooks, i.e. the percentage of (pull) patients that have their start of treatment postponed as the pre-treatment phase cannot be completed in due time.

### Warm-up period and number of replications

**Fig. 4:**
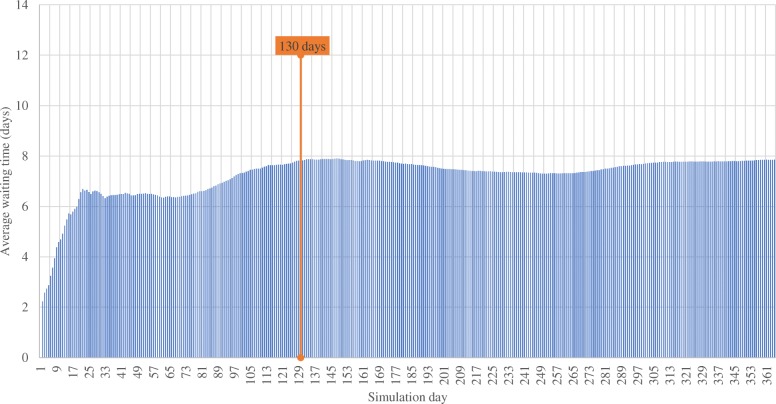
Warm-up analysis: evolution of the cumulative average waiting time over a run of 365 days using 2017 data

Since the model starts in an empty state with no queues and idle resources, we introduced a warm-up period by running the model for one-year data to assess the time needed for the resources to be occupied and the queues filled up. By measuring the evolution of patients’ waiting times over time, the warm-up analysis showed that a steady state is achieved at around 130 days (see Fig. 4). Therefore, during the 130 first simulation days of our computational experiments, output measurements are not included in the results. The 130-day warm-up period runs before the simulation run length of 365 days, which corresponds to the year 2017.

In order to find the proper number of replications, we performed several computational experiments with a different number of replications (*n* = 2,3,4, …) until the relative error of the halfwidth of the confidence interval of the average waiting times ($\overline{x}$) measured across n was sufficiently small (*γ*^′^ < 0.05), according to Eq. (1). Since the sample size (number of replications) is small and thus the real variance is unknown, we use a student’s t-distribution to estimate the confidence interval of $\overline{x}$ for the corresponding number of replications n being tested. The halfwidth of the confidence interval is therefore obtained by ${\boldsymbol{t}}_{\boldsymbol{n}-\mathbf{1},\mathbf{1}-\boldsymbol{\alpha} /\mathbf{2}}\cdotp \frac{\boldsymbol{s}}{\sqrt{\boldsymbol{n}}}$, with s being the variance of the waiting times for n replications, and ***t***_***n*** − **1**, **1** − ***α***/**2**_ being the percentile of the Student-t distribution for n − 1 degrees of freedom at ***t***_**1** − ***α***/**2**_ for a confidence level (1-α). In our experiments, since we consider a 95% confidence level, thus we set α = 0.05.

1 $$ \frac{{\boldsymbol{t}}_{\boldsymbol{n}-\mathbf{1},\mathbf{1}-\boldsymbol{\alpha} /\mathbf{2}}\cdotp \frac{\boldsymbol{SD}}{\sqrt{\boldsymbol{n}}}}{\overline{\boldsymbol{x}}}<{\boldsymbol{\gamma}}^{\prime } $$

By measuring the relative error according to the left-hand side of Eq. (1) for each replication number (*n* = 2,3,4, …), we found that the relative error was smaller than *γ*^′^ = 0.05 for *n* = 15 replications, with a relative error of 0.048. Therefore, we decided to run 15 replications of each computational experiment in our case study.

### Workflow control analysis

To test the impact of increasing the number of patients being scheduled with a pull strategy starting from the baseline case, we gradually added subpopulations of patients based on tumor sites to the current pool of patients being scheduled with a pull strategy. The more complex the pre-treatment process of a patient is, the higher the uncertainty regarding the time needed to complete pre-treatment. Therefore, we started adding patients from the simplest to the most complex tumor types in terms of treatment preparation.

### Scenario analysis

In conjunction with the workflow control analysis, we have investigated the impact of additional interventions that may lead to performance improvements in the NKI. The following scenarios were tested on the baseline case (i.e. with only 40% pull patients):

1. *Spreading consultation slots throughout the week*: We tested the impact of spreading the consultation time slots over the week by setting the same patient arrival mean on every weekday per care trajectory. The overall mean arrival rate, per care trajectory, remains constant.
2. *No pre-allocated time slots for CT:* We tested the impact of removing the pre-allocated slots from the CT tactical plan, by allowing full flexibility to schedule any patient in any available slot as they arrive.
3. *Balancing doctor availability for contouring*: We re-arranged the doctors’ agenda such that each doctor is available for contouring for (at least) 2 h a day, while working the same number of hours per week.
4. *P3 planners can process lung and chest wall patients:* We studied the influence of having P3 planners capable of performing treatment planning of lung and chest wall patients (16.4% increase), in addition to the current tumor sites (rectum, prostate, and breast).
5. *One more full-time P4 planner:* we evaluated the possible gain in waiting times by having one more planning RTT of level P4 (thus capable of performing P4, P3, and P2).

## Results

For model validation, we have compared several outputs of the model for the baseline case with the clinical performance regarding the main KPIs that could be measured in practice for the year 2017 (Table 5). We verify that the total average waiting time (WT) output by the DES model (7.8 days) is very close to the one measured in the actual system, i.e. in the NKI practice (7.9), with the actual system value falling within the 95% confidence interval of the DES model. A similar behavior is observed for the pull and push patient trajectories, with pull patients having lower overall waiting times than average, as in current practice most of these patients are subacute. Regarding the timeliness target fulfillment, the model outputs an average of 85.13 patients breaching their targets, below the value observed in practice (92). Moreover, generated input data, including patient arrival histograms, care content, urgency level and process times, have been compared and found to be consistent with the historical data. The outcomes measured in the actual system and the output values obtained by the model were considered close enough to regard the DES model as a close representation of the actual system behavior, and therefore validated. The final DES model and corresponding outcomes therefore served as the baseline case for running the computational experiments previously described.

**Table 5:** Comparison between the clinical performance and the DES model for validation purposes

Performance metric	Actual system	DES model (95% conf. interval)
Waiting time (total)	7.9	7.8 (7.5, 8.1)
Waiting time (pull)	5.9	5.6 (5.4, 5.9)
Waiting time (push)	9.7	9.7 (9.4, 10.0)
No. patients breaching WT target	92	87.7 (68.1, 107.4)

**Fig. 5:**
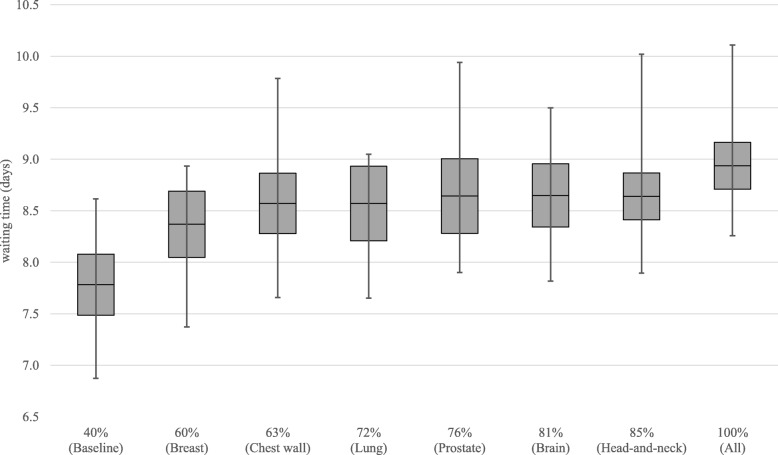
Box plot of the average waiting time (days) for different percentages of patients being scheduled in a pull manner for the workflow control analysis

**Fig. 6:**
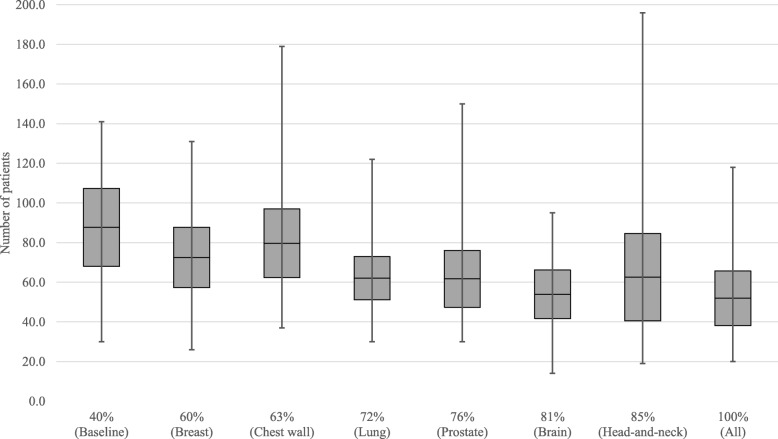
Box plot of the average number of patients starting treatment after the desired waiting time for different percentages of patients being scheduled in a pull manner for the workflow control analysis

**Fig. 7:**
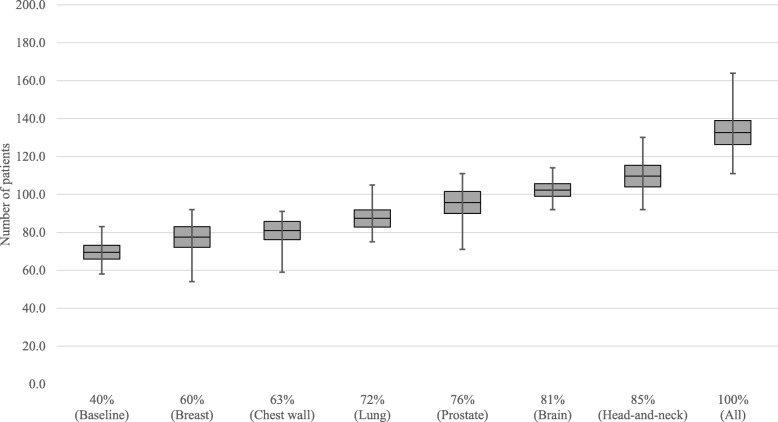
Box plot of the average number of start of treatment rebooks for different percentages of patients being scheduled in a pull manner for the workflow control analysis

Figure 5 shows the effect of increasing the number of pull patients on the overall waiting times. The grey boxes indicate the 95% confidence interval of the average, while the whiskers represent the minimum and maximum values found over the 15 replications. Results show that with the increase of pull patients, the waiting times tend to slowly increase, ranging from 7.8 on the baseline case to an 8.9 maximum, when all patients are scheduled on a pull way. Nevertheless, the addition of some tumor sites like lung or prostate, to a pull strategy, do not impact waiting times considerably. Figure 6 shows the evolution of the number of patients breaching the national waiting time targets: 1 day for acute patients, 10 days for subacute, and 28 days for regular. Overall, the number of breaching patients tends to decrease with the use of a pull strategy. The average number of patients starting treatment after their due date goes down from 87.7 to 51.9, with the maximum topping at 118 patients over all replications when all patients are scheduled on a pull fashion. Figure 7 shows how a pull strategy affects number of first fraction rebooks, i.e. when the pre-treatment workflow cannot be completed before the pre-scheduled date. The more pull patients, the more rebooks occur, with an increase from 69.5 (baseline) to 132.7 (all) in the average number of occurrences.

Table 6 shows the results of the scenario analysis. Balancing the consultation slots had the greatest impact on the performance, by decreasing waiting times from 7.8 to 6.2 days (20.8%) while providing a reduction in the number of patients breaching their waiting time targets from 88 to 23 (74%). Similarly, by not having a pre-allocation of time slots in the CT scanners results show that lower waiting times (17.3%) and fewer patients breaching their targets (57.8%) could be achieved. As for treatment planning, results indicate that performance would modestly improve by either having P3 planners doing lung and chest wall patients (1.6%) or hiring an extra P4 full-time planner (1.4%). Balancing the doctors’ time available for contouring throughout the week has shown not to improve performance, providing the same average waiting time as the baseline case.

**Table 6:** Results of the scenario analysis for the baseline case (i.e. 40% pull patients)

Scenario	Average WT days (95% CI)	# patients breaching WT target (95% CI)	# first fraction rebooks (95% CI)
*Baseline (DES model)*	7.8 (7.5, 8.1)	87.7 (68.1, 107.4)	69.5 (65.9, 73.2)
Spread consultation slots over the week	6.2 (6.1, 6.3)	22.5 (19.0, 26.0)	60.7 (56.4, 65.1)
No pre-allocation for CT	6.4 (6.4, 6.5)	37.1 (31.8, 42.4)	65.6 (62.4, 68.8)
Balance doctor availability for contouring	7.8 (7.5, 8.0)	80.9 (66.1, 95.6)	76.9 (73.4, 80.5)
Increase automated planning by 16.4%	7.7 (7.4, 7.9)	74.2 (61.0, 87.4)	67.5 (62.9, 72.2)
One more full-time P4 planner	7.7 (7.4, 7.9)	77.3 (62.3, 92.4)	64.3 (60.3, 68.2)

## Discussion

We have developed a discrete-event simulation model to assess the optimal balance between two different strategies for patient scheduling in RT: pull (schedule at first consultation) and push (schedule after treatment planning), based on the actual system data of the NKI. Results showed that increasing the pull strategy from 40 to 100% reduces the number of patients starting treatment after the WT target date from 87.7 to 51.9 (Fig. 6), on average. By setting a start of treatment right at the beginning of the process, the control over the work-in-progress obviously increases and there is a lower risk of having delayed patients. This can be achieved at a cost of a maximum of 1 day increase in the average waiting times (Fig. 5). A push strategy, by allowing work to flow continuously throughout the RT chain, provides up to 1.1 days reduction in the average waiting times. However, in moments of high workload and/or reduced staff availability while using a push system, some patients may have to wait longer than desired and consequently breach their WT target date, which can be mitigated by a pull strategy. As expected, the percentage of first appointment rebooks gradually increases with a pull strategy, due to non-completion of the pre-treatment phase on time to a maximum of 2.7% (Fig. 7). Moreover, we have found that applying a pull strategy for certain tumor sites has greater impact on performance than for others. For instance, by adding prostate, brain and head-and-neck patients to the pull group, we verified that waiting times remained constant while the number of breaching patients slightly decreased. This may indicate that there is enough capacity in the department to accommodate these patients working on a pull strategy without increasing waiting times. In fact, the process of increasing the number of patients working on a pull fashion can be gradual. For instance, by scheduling all breast patients in addition to the baseline case, thus increasing the total number of pull patients from 40 to 60%, may allow achieving a 17.3% decrease on patients breaching the waiting time targets, with an increase on the average waiting time (6.4%) and the number of first appointment rebooks (11.5%).

A scenario analysis of possible interventions performed on the baseline case (40% pull patients) has shown that distributing consultation time slots evenly throughout the week has the highest impact on the measured performance. As shown in Table 6, by spreading consultations slots evenly over the week and thus keeping workload less variable throughout the chain, average waiting times can potentially decrease from 7.8 to 6.2 days. Although we understand that this may not be straightforward to implement due to the complex doctor schemes and busy agendas, it is an insight that may encourage decision makers to strive for consultation slots spread throughout the week as much as possible for each specialty. In addition, by not having pre-allocated time slots for CT scheduling the average waiting times and number of patients breaching the targets can potentially decrease by 12.7 and 57.8%, respectively (see Table 6). However, since most of the allocated time slots are dedicated to acute and bone metastasis patients, the impact on delays of these patient types would need to be further explored before an actual implementation. Our findings also showed that spreading the availability of doctors to perform contouring over the week does not increase performance, suggesting that the current doctors’ agenda is well synchronized with the patient throughput for contouring. Moreover, our study showed that the increasing the number of planning RTTs does not improve performance significantly when compared to other scenarios, as the addition of an extra full-time RTT with the highest skill level of planning provided a marginal decrease of 1.4% in waiting times and 11.9% in the number of patients breaching the WT targets. Similarly, we found that upgrading the skill level of P3 planners to perform lung and chest wall patients did not impact results considerably from a logistics point of view.

Despite all the insights obtained with the DES model, there are a few limitations to our simulation study. The model is not able to fully capture the behavior of clinicians, as they may for example stay at work longer than expected to finalize certain tasks and avoid delaying the process of more urgent cases or skip certain meetings to do contouring when their clinical workload is high. Given the lack of clinical data regarding these situations, we overlook this possibility in the model. Moreover, each treatment plan needs to be checked and approved by a medical physicist before the first fraction is delivered. However, in the NKI a medical physicist is called by the planning RTT right after completion of the treatment plan. Therefore, there is no delay due to this step. In addition, the treatment plan may need to be improved or modified as a result of the medical physics check, thus requiring extra time to complete the treatment planning phase. We have overlooked these situations in our model as they account for less than 1% of the cases.

## Conclusions

A 100% pull strategy, in which patients are scheduled a start of treatment right after consultation, provides increased predictability on the fulfillment of waiting time targets in detriment of a small increase in the average waiting times when compared to a push strategy. These findings are useful to support policy making in RT regarding their workflow control strategies and help RT centers achieve a desired service level within their resource constraints. Some centers may accept having slightly longer waiting times if that means having their patients informed about the start date for treatment date right at consultation, thus decreasing the discomfort and psychological distress associated with waiting for a date to start treatment. Moreover, DES has proved to be a powerful tool that provides an overview of the actual system and can help RT managers find bottlenecks and opportunities for performance improvement with recourse to visualization tools. Managerial interventions can be tested with little effort after a valid and robust model has been constructed, and the consequences of alternative input parameters can be quickly estimated.

As a follow up of this study, we want to implement and test extending the number of patients being scheduled in a pull way in the RT department of the NKI (e.g. all breast patients) and perform a pre-post performance evaluation to verify whether our theoretical results hold in practice. Furthermore, as the modeled processes and the patient mix are standard among RT centers, the proposed model can also be applied to other centers with a similar workflow and resource schemes.

## Supplementary information

**Additional file 1.** List of care plans in the NKI (2017) and corresponding data used for model inputs

## Data Availability

The datasets used and/or analyzed during the current study are available from the corresponding author on reasonable request.

## Abbreviations

DES: Discrete-Event Simulation
EDD: Earliest Due Date
IPP: Image post-processing
KPI: Key Performance Indicator
LINAC: Linear Accelerator
NKI: Netherlands Cancer Institute
OR: Operations Research
RT: Radiotherapy
RTT: Radiation Therapy Technologist
WT: Waiting Time

## Footnotes

1 time between referral and start of treatment
