# Supplementary material for: Improving workflow control in radiotherapy using discrete-event simulation
Source: BMC Med Inform Decis Mak. 2019 Oct 24;19:199. doi: 10.1186/s12911-019-0910-0 (PMC6814107; doi:10.1186/s12911-019-0910-0)
Supplement: Supplementary file 1 — Additional file 1. List of care plans in the NKI (2017) and corresponding data used for model inputs [file 12911_2019_910_MOESM1_ESM.pdf]

Additional file 1: List of care plans in the NKI (2017) and corresponding data used for model inputs

Care plan	Tumor site	# patients	# acute	# subacute	# regular	# patients with planned delay	Prob. P2	P2 time (min)	P3 time (min)	P4 time (min)
Anus +/- inguinal lymph node	Others	22	0	0	22	1	0.05	60	-	480
Adrenal Stereotaxic	Others	7	0	0	7	0	0.00	-	-	420
Bladder	Others	43	0	2	41	2	0.02	60	-	240
Bladder (partial)	Others	22	0	0	22	5	0.00	-	-	240
Chest wall	Chest wall	45	0	0	45	20	0.02	60	-	120
Chest wall+Axilla	Chest wall	114	0	0	114	57	0.85	90	-	240
Chest wall+Axilla+Parasternal	Chest wall	15	1	1	13	7	0.60	120	-	240
Chest wall+Parasternal	Chest wall	3	0	0	3	2	0.33	60	-	240
Chest wall (bsu)	Chest wall	2	0	1	1	1	1.00	60	-	-
Bone metastasis	Bone met.	1119	53	1006	60	32	0.93	60	-	300
Bone metastasis Stereotaxic	Bone met.	56	0	1	55	1	0.14	60	-	420
Brachy Bladder	Others	15	0	0	15	0	0.00	-	-	-
Brachy Cilinder	Others	21	0	0	21	0	0.05	60	-	-
Brachy Intrauterine	Others	27	0	0	27	0	0.00	-	-	-
Brachy IOBT	Others	1	0	0	1	0	0.00	-	-	-
Brachy Oesophagus	Others	1	0	0	1	0	0.00	-	-	-
Brachy Prostate	Others	6	0	0	6	0	0.00	-	-	-
Uterus	Others	43	0	2	41	4	0.02	60	-	360
Endometrium	Others	17	0	0	17	1	0.06	60	-	360
Neck (bsu)	Others	9	0	8	1	1	1.00	60	-	-
Brain 1 fraction	Brain	140	0	10	130	2	0.14	60	-	360
Brain several fractions	Brain	94	0	5	89	3	0.09	60	-	360
Brain (whole)	Brain	166	2	134	30	8	1.00	60	-	-
Brain Electrons	Others	6	0	0	6	1	0.17	60	-	360
Lymph node Stereotaxic	Others	22	0	0	22	3	0.00	-	-	300
Head-and-neck	Head&neck	237	0	2	235	21	0.00	60	-	480
Head-and-neck (palliative)	Head&neck	4	0	0	4	1	0.25	60	-	480
Larynx 2vs	Others	2	0	0	2	0	0.00	-	-	480
Liver	Others	34	0	1	33	1	0.03	60	-	600
Lung (palliative)	Lung	65	1	51	13	5	0.42	60	-	690
Lung	Lung	283	1	8	274	15	0.08	60	-	690
Lung (bsu)	Lung	50	0	46	4	2	0.90	60	-	690
Lung Stereotaxic	Lung	239	0	2	237	11	0.01	60	-	420
Lymphoma	Others	43	0	2	41	6	0.07	60	-	300
Lymphoma (bsu)	Others	18	0	3	15	2	0.78	60	-	300
Stomach	Breast	14	1	1	12	2	0.21	60	-	360
Breast	Breast	777	0	7	770	276	0.03	60	120	120
Breast+Axilla	Breast	180	0	0	180	69	0.63	90	240	240
Breast+Axilla+Parasternal	Breast	19	0	0	19	9	0.53	120	240	240
Breast+Parasternal	Others	1	0	0	1	0	1.00	60	120	120
Esophagus	Others	78	1	5	72	9	0.09	60	-	360
Esophagus (palliative)	Others	22	1	18	3	1	0.55	60	-	360
Axilla (virtual)	Others	5	0	4	1	0	0.60	60	-	300
Orbit (eye socket)	Others	1	0	1	0	0	0.00	-	-	480
Ovaries	Others	5	0	2	3	1	0.00	-	-	360
Others	Others	186	0	138	48	18	0.07	60	-	300
Others (bsu)	Others	62	1	56	5	2	0.87	60	-	300
PAO (+/- iliac single-sided)	Others	1	0	0	1	0	0.00	-	-	240
Penis	Others	11	0	1	10	0	0.00	-	-	480
Prostate	Prostate	243	0	1	242	12	0.01	60	150	150
Prostate+Pelvic lymph nodes	Prostate	61	0	0	61	8	0.00	-	420	420
Prostatic bed	Prostate	36	0	0	36	2	0.00	-	300	300
Prostatic bed+Pelvic lymph nodes	Prostate	25	0	0	25	5	0.00	-	420	420
Rectum / Sigmoid	Others	88	0	4	84	6	0.02	60	300	300
Rectum 13 x 3 Gy	Others	6	0	5	1	0	0.00	-	300	300
Rectum 5 x 5 Gy	Others	57	1	0	56	2	0.00	-	300	300
Sarcoma abdominal/thoracic wall	Others	13	0	0	13	4	0.00	-	-	240
Sarcoma extremity	Others	31	0	4	27	2	0.03	60	-	480
Sarcoma retroperitoneal	Others	9	0	0	9	0	0.00	-	-	240
Spinal cord	Others	37	0	1	36	4	0.16	60	-	480
Vagina	Others	5	0	0	5	1	0.00	-	-	360
Vulva +/- inguinal lymph nodes	Others	9	0	0	9	2	0.00	-	-	420
**Total**		**4973**	**63**	**1533**	**3377**	**650**				
**% of patient population**			**1.3%**	**30.8%**	**67.9%**	**13.1%**				
